# Manufacturing Cells for Clinical Use

**DOI:** 10.1155/2016/1750697

**Published:** 2016-06-12

**Authors:** Mark L. Weiss, Mahendra S. Rao, Robert Deans, Peter Czermak

**Affiliations:** ^1^Department of Anatomy and Physiology, Midwest Institute for Comparative Stem Cell Biology, Kansas State University, Manhattan, KS 66506, USA; ^2^The New York Stem Cell Foundation, New York, NY 10023, USA; ^3^Rubius Therapeutics, Cambridge, MA 02139, USA; ^4^Institute of Bioprocess Engineering and Pharmaceutical Technology, University of Applied Sciences Mittelhessen, 35390 Giessen, Germany; ^5^Faculty of Biology and Chemistry, Justus Liebig University Giessen, 35390 Giessen, Germany

## 1. Introduction

**Figure 1:**
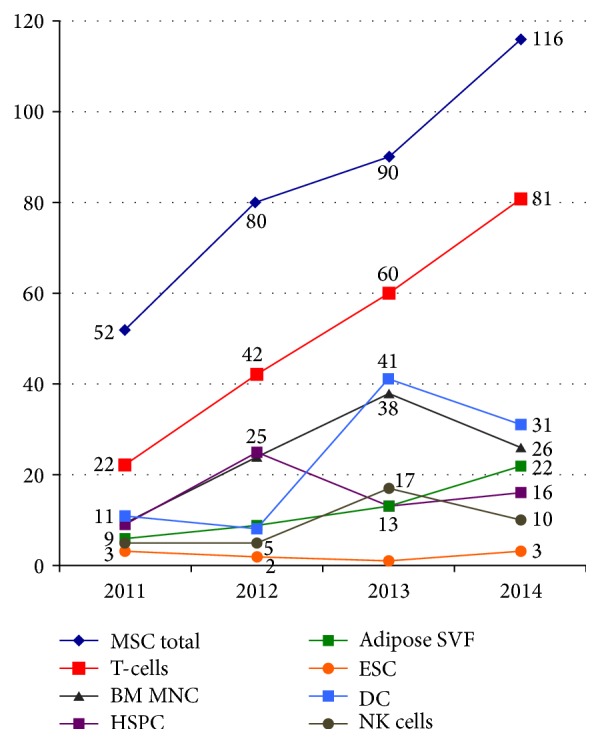
Number of stem cell therapy trials by cell type worldwide (reprinted from [9]).

The growth in the number of registered clinical trials indicates that there is a need for cells for many types of cell therapy.  Figure 1, which is reprinted from the excellent blog maintained by Alexi Bersenev, shows that the cell type used in most clinical trials worldwide is the mesenchymal stromal cell (MSC). The MSC type requires* in vitro* expansion to reach a clinical dose and thus there is a desire to optimize and standardize processes and procedures for MSC manufacture specifically for clinical use.

When considering MSC, there are “issues” associated with manufacturing which warrant special consideration. Some of these issues are associated with the identification of the MSC source used for therapeutic cells because MSCs can be isolated from different tissues. For example, they can be isolated from fat, bone marrow, or fetal tissues such as placenta or umbilical cord. For autologous use, MSCs from fat have received attention. MSCs can be isolated from donors of different age or sex. Finally, the health status of the donor may affect MSC function. These factors may affect MSC isolation, expansion capability, function, and/or survival after transplantation. Thus, MSCs from one tissue may be superior to MSCs from another for a particular clinical application. However, if one cannot expand the MSCs to a therapeutic dose, it moots the point.

Much attention has focused upon manufacturing or expanding MSCs for clinical use. There is a lack of standardization with MSC characterization, as recently reviewed [1, 2]. Some researchers suggested that “standardized” MSCs be derived, expanded, and provided to the research community as a means of addressing the differences in MSC characterization found between laboratories [2, 3]. Other researchers suggested that standardization of the characterization methods and tools is needed [1]. When one considers that MSC manufacturing and passaging contribute to the differences mentioned above, for example, MSCs isolated from different passages [4, 5] or from donors of different tissues, ages, or health status reflecting the inherent biological variability, MSCs might defy standardization [2, 6–8]. As a first step, it might be easiest to establish standardized characterization tools and protocols or perhaps to use a certification procedure to make the characterization of MSCs between laboratories more uniform.

In this issue, eight articles address different aspects of manufacturing cells for cell therapy. These articles can be broken down into two groups. The first group deals with scale-up and manufacturing of MSCs and consists of five papers (those of J. R. Smith et al., F. Petry et al., D. Salzig et al., R. J. Emnett et al., and V. Jossen et al.). The second group deals with application and optimizing of cell therapy and consists of three papers (those of N. E. Rekittke et al., S. Zhou et al., and J. E. V. Meraz et al.).

## 2. Manufacturing MSCs for Clinical Use

J. R. Smith et al. focused efforts to standardize and optimize the isolation of MSCs from the umbilical cord. They developed a process to extract cells using Miltenyi C-tubes coupled with enzymatic digestion with minimal dissection that resulted in 10x more MSCs than their previously described method, while also reducing contamination risk. They expanded the MSCs in xenogeneic-material-free conditions using 10% pooled human platelet lysate to supplement the growth medium. MSCs produced using their new method met the minimal definition of MSCs set by ISCT's MSC working group. They also had one unexpected finding: a higher yield of MSCs from cords derived from natural births compared to Caesarian-section births. They did not identify the mechanism responsible. Taken together, their findings provided a more streamline extraction method and an optimized expansion protocol suitable for translation to clinical manufacturing. The main limitation for clinical translation is the use of enzymatic digestion.

The contribution by F. Petry et al. involves the growth of MSCs in three dimensions (3D) with scale-up to clinical manufacturing in a stirred tank bioreactor. They started the scale-up by identifying microcarriers that would be suitable to the umbilical cord MSCs in small-scale spinner flasks. Interestingly, the umbilical cord MSCs preferred a plastic microcarrier, as opposed to the treated glass microcarrier that telomerase immortalized bone marrow MSCs prefer [10–12]. It appears that MSCs from different tissue sources have different attachment requirements. Or perhaps the differences are due to the use of pooled human platelet lysate as a medium additive in F. Petry et al.'s paper compared to fetal bovine serum used to expand the bone marrow-derived MSCs. After identifying the best microcarrier, F. Petry et al. tested seeding protocols and feeding strategies in spinner flasks. The paper culminated in scale-up to 2.5 L in a Millipore Mobius single use stirred tank bioreactor. This paper identified scaling factors and set the stage for more highly refined optimization and an SOP for MSC expansion. The MSCs produced in 3D met the minimal definition of MSCs and were not obviously different from the MSCs expanded in 2D in their companion paper (J. R. Smith et al.).

The paper by D. Salzig et al. involved the optimization of MSC expansion in chemically defined medium (CDM) for GMP manufacturing. The use of a chemically defined medium might eliminate the need for batch-to-batch comparison and validation of medium which employs biologics. D. Salzig et al. compared the growth of both an immortalized MSC line and primary MSCs from bone marrow or adipose tissues in CDM following various surface treatments of the substrate to enhance attachment. They found a difference in the surface attachment requirements between the MSC types. For example, bone marrow-derived MSCs did not attach rapidly in CDM, compared to immortalized MSCs or adipose-derived MSCs that attached in 2–5 hrs. Immortalized MSCs attached readily to specialized tissue culture plastic in CDM and grew as well as or better than MSCs seeded onto standard tissue culture plastics in serum containing medium. In contrast, surface coating with collagen type IV or fibronectin, but not laminin, was important for bone marrow-derived MSC's attachment and growth. Interestingly, for the bone marrow MSCs, D. Salzig et al. were unable to achieve MSC expansion equal to serum containing medium. Next, they evaluated the detachment efficiency of four different enzymes (trypsin, accutase, prolyl-specific peptidase (PsP), and collagenase) on bone marrow-derived MSCs and immortalized MSCs. Again, they found differences between MSCs source and the type of medium on the detachment efficiency. For immortalized MSCs, CDM reduced the efficiency to detach, compared to serum containing medium, and collagenase was inferior to trypsin, accutase, and PsP. In contrast, all four enzymes performed equally well for detaching bone marrow-derived MSCs grown in serum containing medium, with little or no effect of surface treatment. These results indicate that derivation of SOPs for MSC expansion and harvest might need to be customized for each type of medium used and MSCs tissue source. The data in this paper argues against the notion that standardized conditions for MSCs can be generalized across tissue source.

R. J. Emnett et al. developed a GMP-compliant method to isolate mesenchymal stromal cells (MSCs) from the umbilical cord. Since many of the currently described methods use enzymatic digestion to extract cells from the cord and since that step introduces xenogeneic reagents, R. J. Emnett et al. were determined to extract MSCs following mechanical disruption of the cord using a tissue homogenizer and they compared the yield to that obtained following collagenase digestion. Next, they expanded MSCs in medium supplemented with 10% pooled human platelet lysate or 20% fetal bovine serum. They found that mechanical disruption produced an identical yield as collagenase extraction at the initial isolation step. However, following expansion, fewer cells were produced after mechanical dissociation compared to enzymatic extraction. Mechanical extraction may have introduced higher levels of cellular stress and this could have produced the observed slower expansion at both early passage (P1–P3) and later passage (P7–P9). Mechanical dissociation impaired colony-forming efficiency of MSCs compared to MSCs enzymatically extracted when MSCs were expanded in pooled human platelet lysate, also suggesting that MSCs isolated by mechanical disruption were stressed. R. J. Emnett et al. calculated that they could theoretically reach their manufacturing goals of 30 billion MSCs within approximately one month of culture for either method. Their method is easily scaled up, requires minimal manipulation of the cord tissues prior to extraction within a closed system, and produces MSCs that meet the ISCT definition for surface marker expression and trilineage differentiation. While additional characterization work would be needed to validate that MSCs expanded following mechanical extraction are bioequivalent to MSCs expanded following enzymatic isolation methods, the simplicity of this method is encouraging and easily standardized between laboratories.

V. Jossen et al. examined the expansion of human adipose-derived MSCs on microcarriers for manufacturing scale-up in both stirred tank bioreactor and a bag (wave-mixer type) bioreactor. Their intent was to investigate the relationship between impeller speed and shear stress and to minimize the aggregation of microcarriers which is thought to result in limitations of mass transfer and heterogeneous distribution of cells on the carriers. Further, aggregation of carriers may interfere with the use of computational methods to model scale-up results. Here, V. Jossen et al. used adipose-derived MSCs from a single donor. These MSCs were expanded in Lonza specialty medium supplemented with 5% fetal bovine serum using polystyrene microcarriers. The authors found the largest expansion factor at impeller speeds that were slower than the suspension speed (N_s1_), at a speed they term N_siu_. It should be noted that N_s1_ (sometimes called N_SJ_) is used in the industry as an accepted impeller speed since this is the minimal speed to suspend the carriers in the fluid column, thereby maximize mass transfer, and minimize the possibility of heterogenous distribution of cells on the carriers. The authors show enhanced growth rate and therefore expansion factor at a slightly slower impeller speed (N_s1u_) and suggest that the lower shear stress on the cells may explain the differences. One confounding factor to their experiment was that aggregates of MSCs and microcarriers occurred by the end of the culture period. Thus, there are still issues to be resolved. V. Jossen et al. performed experiments expanding MSCs in a bag type of bioreactor to evaluate the differences between N_s1_ and N_s1u_ between the two types of bioreactors. They evaluated the maximum power and the shear stress in the wave type bioreactor, which provides a rhythmic periodic stress as opposed to the constant stresses provided in stirred tank bioreactors. Finally, they performed a proof-of-concept expansion of MSCs to compare yields between stirred tank and wave type bioreactors. While propagation of MSCs was possible in both bioreactors, the yield in the wave type bioreactor was three times lower than the stirred tank system. In addition, the wave type bioreactor produced larger aggregates, up to 6 mm in size. Their results confirm earlier reports that N_s1u_ produces superior propagation of MSCs in stirred tank bioreactors and that stirred tank bioreactors are superior for MSC expansion compared to wave type bioreactors. These data confirm that shear stress and aggregate formation negatively affect MSC expansion. When this paper is taken in the context of others within this special edition, one wonders whether these data can be extended to other media conditions, such as a chemically defined medium as discussed in R. J. Emnett et al.'s paper, or whether these data can be extended to other MSC sources (as in D. Salzig et al.'s paper). Using mathematical modeling to optimize MSC manufacturing is a new topic that will be refined as additional results identify the key variables.

### 2.1. Application and Optimizing of Stem Cell Therapy

When treating many forms of hematological cancers or solid tumors, hematopoietic stem cell transplants reconstitute hematopoiesis after myeloablative therapy to reduce tumor burden. Mobilized peripheral blood progenitor cells collected by aphaeresis have become a common means to treat myeloablative therapy in such patients, replacing even bone marrow transplant due to the rapid recovery following transplantation. J. E. V. Meraz et al. compared three different stem cell mobilization methods in children with malignant cancers to evaluate which was best for hematopoietic stem cell mobilization. The three methods were cyclophosphamide (CFA) and hematopoietic growth factor G-CSF with aphaeresis beginning when white blood count (WBC) exceeded 1 × 10^9^/liter (group A), CFA with G-CSF with aphaeresis beginning when WBC exceeded 10 × 10^9^/liter (group B), or G-CSF alone for 4 days followed by aphaeresis beginning on the fifth day (group C). In each case, aphaeresis continued until the target dose of CD34+ cells and MNCs reached 2 × 10^8^/kg and 4 × 10^8^/kg, respectively. Adverse events, recorded as hospitalization instances due to neutropenia, were recorded in the CFA-treated groups (groups A and B), but not in the G-CSF only group (group C). While group B appears to be superior to group A for mobilization (total MNC and CD34+ cell number), there were no differences between groups B and C. J. E. V. Meraz et al. concluded that CFA and G-CSF mobilization when WBCx exceeds 10 × 10^9^/kg had similar efficacy to mobilization with G-CSF alone, but G-CSF alone had fewer adverse events. Since their research did not follow through with transplantation outcomes, it is unclear whether the hematopoietic transplant of mobilized blood from G-CSF alone (group C) would reconstitute the patients more rapidly than those patients receiving the combined CFA and G-CSF. J. E. V. Meraz et al.'s results suggest that the G-CSF protocol is currently the best preparation for hematopoietic stem cell transplantation in children.

N. E. Rekittke et al. provided a review of the state of the art of regenerative medicine therapy for type 1 diabetes mellitus. Their review focuses on pancreatic islet transplantation and on undifferentiated MSC transplantation and transdifferentiated MSCs to endoderm or pancreatic progenitor cells. Pancreatic islet transplants have been evaluated in clinic studies in the USA and abroad. N. E. Rekittke et al.'s review covers the major progress in islet transplantation trials through three iterations: improvements in cadaveric islet processing to reduce damage and refinements of immune suppression in the recipient represent the important stages of progress. According to N. E. Rekittke et al.'s review, following the latest procedures, the majority of diabetic patients who receive islet transplantation can expect to become insulin-independent for up to five years. N. E. Rekittke et al. discussed unsolved issues of human islet transplants including the blood-mediated inflammation that impairs islet function and survival and the need to develop improved islet isolation protocols which enhance islet function and survival and reduce demand for cadaveric material to meet demand. In addition to reviewing translation research on islet transplantation, N. E. Rekittke et al. discussed the use of MSCs for type I diabetes therapy. This review indicates that progress in islet isolation and immune suppression of the recipient is responsible for the great progress in pancreatic islet transplantation. If the improved immune suppression regimes developed for pancreatic islet cell transplantation also can enable other allogeneic cell transplantation, this work may have a tremendous impact on the patients needing tissue transplantation.

Closing out this special issue on manufacturing cells for cell therapy, S. Zhou et al. provided a state-of-the-art review on the use of stem cell therapy to treat stress urinary incontinence (SUI). SUI may affect more than 200 million people worldwide and disproportionately affects women at a ratio of 3 : 1. Pregnancy and having a child by vaginal birth are risk factors for SUI due to functional loss of levator ani muscles. Similarly, men who have radical prostatectomy are at increased risk for SUI. The most common and effective treatments of SUI are bulking agent injections to augment levator ani function. These bulking agents are associated with a variety of adverse events suggesting that an alternative approach, such as the use of stem cells, may assist with regeneration or repair of levator ani and be effective for treating SUI. S. Zhou et al. reviewed the preclinical literature which supports the use of various autologous stem cell sources, including muscle-derived stem cells, adipose-derived MSCs, and bone marrow-derived MSCs, for treating SUI. This work is still in the preclinical stage, mostly, and a few safety studies were discussed. While injection of stem cells as bulking agents for levator ani muscle is one direction for SUI treatment, S. Zhou et al. discuss the tissue engineering approach, too, which involves the use of scaffolds seeded with autologous stem cells. Work to date includes preclinical testing in the rat SUI model as an alternative to surgical tape. Limitation of both approaches included the need to improve cell survival after transplantation. This limitation was identified by the review by N. E. Rekittke et al. also in this issue, which indicated that the rapid progression and increased therapeutic impact after islet transplantation in type I diabetes were coupled with improving the quality of transplanted cells and improved immune suppression. Since stem cells transplanted to treat SUI were of autologous origin, it is likely that larger improvements will be obtained by improving the cell preparation and transplantation to reduce cell stress and improve survival after transplantation. While one might expect that immune suppression would not be required in the autologous transplantation setting, it is likely that transient immune suppression is also likely to improve outcomes in this situation, too.

## 3. Conclusions

Cellular manufacturing for clinical applications is moving forward rapidly due to pressing need created by the hundreds of ongoing clinical trials and due to the research results providing the fundamental knowledge about barriers and how to overcome them. In this special issue, topics ranged from isolation and manufacturing of MSCs to critical reviews of the application of cell therapy for treating type I diabetes using islet transplantation or treatment of stress induced urinary incontinence. From reviewing the issues, a key take-home message is that it is not enough to produce cells in sufficient numbers. Equally important is to produce cells that survive and function following transplantation. In summary, the manufacture of clinical doses of robust functional cells is one key piece to cell therapy; the other key is the preparative regime given to the patient to enable engraftment and function of cells. Looking back in history, we find this to be a recurrent theme from hematopoietic stem cell transplantation. Understanding how to manufacture cells for clinical purpose is fundamental to improving outcomes and provides a starting point to evaluate patient preparative regimens.

*Mark L. Weiss* *Mark L. Weiss* *Mahendra S. Rao* *Mahendra S. Rao* *Robert Deans* *Robert Deans* *Peter Czermak* *Peter Czermak*
